# Loss of *plakoglobin *promotes decreased cell-cell contact, increased invasion, and breast cancer cell dissemination *in vivo*

**DOI:** 10.1186/bcr3201

**Published:** 2012-05-25

**Authors:** Ingunn Holen, Jacob Whitworth, Faith Nutter, Alyson Evans, Hannah K Brown, Diane V Lefley, Ivana Barbaric, Mark Jones, Penelope D Ottewell

**Affiliations:** 1 Academic Unit of Clinical Oncology, University of Sheffield, Medical School, Beech Hill Road, Sheffield, S10 2RX, UK; 2 Centre for Stem Cell Research, University of Sheffield, Alfred Denny Building, Western Bank, Sheffield, S10 2TN, UK

## Abstract

**Introduction:**

The majority of deaths from breast cancer are a result of metastases; however, little is understood about the genetic alterations underlying their onset. Genetic profiling has identified the adhesion molecule *plakoglobin *as being three-fold reduced in expression in primary breast tumors that have metastasized compared with nonmetastatic tumors. In this study, we demonstrate a functional role for *plakoglobin *in the shedding of tumor cells from the primary site into the circulation.

**Methods:**

We investigated the effects of *plakoglobin *knockdown on breast cancer cell proliferation, migration, adhesion, and invasion *in vitro *and on tumor growth and intravasation *in vivo*. MCF7 and T47D cells were stably transfected with miRNA sequences targeting the *plakoglobin *gene, or scramble vector. Gene and protein expression was monitored by quantitative polymerase chain reaction (qPCR) and Western blot. Cell proliferation, adhesion, migration, and invasion were measured by cell counting, flow cytometry, and scratch and Boyden Chamber assays. For *in vivo *experiments, *plakoglobin *knockdown and control cells were inoculated into mammary fat pads of mice, and tumor growth, shedding of tumor cells into the bloodstream, and evidence of metastatic bone lesions were monitored with caliper measurement, flow cytometry, and microcomputed tomography (μCT), respectively.

**Results:**

*Plakoglobin *and γ-catenin expression were reduced by more than 80% in all knockdown cell lines used but were unaltered after transfection with the scrambled sequence. Reduced *plakoglobin *resulted in significantly increased in MCF7 and T47D cell proliferation *in vitro *and *in vivo*, compared with control, with significantly more tumor cells being shed into the bloodstream of mice bearing *plakoglobin *knockdown tumors. In addition, *plakoglobin *knockdown cells showed a >250% increase in invasion through basement membrane and exhibited reduced cell-to-cell adhesion compared with control cells.

**Conclusion:**

Decreased *plakoglobin *expression increases the invasive behavior of breast cancer cells. This is the first demonstration of a functional role for *plakoglobin*/γ-catenin in the metastatic process, indicating that this molecule may represent a target for antimetastatic therapies.

## Introduction

Breast cancer is a highly metastatic disease, and once metastases are established, this condition becomes incurable. In 65% to 75% of patients with advanced breast cancer, skeletal involvement is present, with median survival rates of approximately 2 years after initial diagnosis of bone metastasis [1]. However, little is understood about the genetic alterations that are responsible for changing a nonmetastatic tumor into a tumor with a metastatic phenotype. Identification of these genes and their functional significance is therefore essential for the development of novel therapeutic regimens.

Genetic profiling has identified the adhesion molecule *plakoglobin *as being the gene most significantly altered in breast cancer that has metastasized to bone and lymph node compared with nonmetastatic tumors; metastatic tumors showed a threefold reduction in expression of this gene [2]. Furthermore, expression of *plakoglobin *is reduced on autocrine production of human growth hormone, which is associated with increased breast tumor cell invasion *in vivo *[3]*. Plakoglobin *resides on the same locus as the *BRCA1 *gene, 17q12-17q21, and patients showing loss of heterozygosity of *BRCA1 *also show loss of heterozygosity of *plakoglobin *[4,5]. Patients with this mutation have an 80% chance of developing breast cancer, further suggesting a role for *plakoglobin *in the etiology of breast cancer [6].

For metastases to develop, tumor cells must first acquire a motile phenotype and migrate toward the basement membrane. Breakdown of the extracellular matrix then allows the cells to invade the surrounding tissue and enter the bloodstream or lymphatic system. Once in the circulation, cells must evade the host immune system before exiting the vasculature by adhering to the endothelium of the target site. Finally, tumor cells implant at a secondary site, forming a metastases [7]. Therefore, genetic alterations that lead to disruption of cell-cell and cell-matrix interactions may play a role in the initiation of cancer metastases, facilitating tumor-cell dissemination from the primary site into the circulation.

*Plakoglobin *encodes for the cell-adhesion protein γ-catenin [8,9]. In conjunction with β-catenin, γ-catenin links e-cadherin to the actin cytoskeleton, forming intercellular cadherin-catenin complexes, a key part of the extracellular matrix [10]. In this study, we aimed to establish whether *plakoglobin *has a functional role in the metastatic cascade in breast cancer. We investigated the effects of reducing *plakoglobin *expression on the invasive properties of breast cancer cells by using miRNA technology. We have designed small ssRNA sequences of approximately 22 nucleotides in length that trigger gene silencing through specific cleavage and RNA degradation to target *plakoglobin *exclusively [11,12]. We compared the effects of reduced *plakoglobin *expression in weakly metastatic MCF7 and nonmetastatic T47D breast cancer cells on growth, adhesion, migration, and invasion *in vitro *[13-16]. In addition, we analyzed tumor growth and intravasation from the mouse mammary fat pad into the circulation *in vivo*. Our data show that loss of *plakoglobin *promotes the prometastatic phenotype of breast cancer cells, indicating that this molecule may be a potential prognostic factor of increased risk of cancer progression as well as a therapeutic target.

## Materials and methods

### Generation of *plakoglobin *knockdown cell lines

Pre-miRNA expression cassettes were designed to target the *plakoglobin *gene by using the BLOCK-iT Pol II miR RNAi Expression vector kits and manufacturer's instructions. In brief, complementary DNA oligos containing four-nucleotide overhangs were designed by using the RNAi Designer online tool (Invitrogen, Carlsbad, CA, USA) and synthesised by using the following primer pairs: **2 **= Forward (5' to 3'): TGC TGA TGA GAT GCA CAA TGG CCG ACG TTT TGG CCA CTG ACT GAC GTC GGC CAG TGC ATC TCA T Reverse (5' to 3'): CCT GAT GAG ATG CAC TGG CCG ACG TCA GTC AGT GGC CAA AAC GTC GGC CAT TGT GCA TCT CAT C. **3 **= Forward (5' to 3'): TGC TGA TTG CTG GGA CAC ACG GAT AGG TTT TGG CCA CTG ACT GAC CTA TCC GTG TCC CAG CAA T Reverse (5' to 3'): CCT GAT TGC TGG GAC ACG GAT AGG TCA GTC AGT GGC CAA AAC CTA TCC GTG TGT CCC AGC AAT C. Double-stranded oligos were then cloned into pcDNA6.2-GW/EmGFP-miR expression vectors by using T4 DNA Ligase before being transformed into One Shot TOP10 Competent *Escherichia coli*. Transformed *E. coli *was plated onto LB agar, Miller (Sigma-Aldrich, Poole, UK) containing 100 μg/ml nucleoside antibiotic (Blasticidin; Invitrogen). To check accuracy of cloning and transformation DNA from five individual Blasticidin-resistant colonies per original double-stranded oligo was purified by using PureLink HQ Mini Plasmid purification Kit (Invitrogen), sequenced in house by using the random shotgun method by the Genomics core facility, University of Sheffield, as previously described; data were analyzed by using Finch TV software and data cross referenced to the *plakoglobin *sequence on Genbank [Genbank: AC000149] [17,18]. To enable lentiviral transfection, the pre-miRNA expression cassettes were then transferred to pLenti6/V5-DST destination vector (as described in BLOCK-iT Lentiviral PolII miRNAi Expression System manual, available online) [19]. Pre-miRNA cassettes and ViraPower (Invitrogen) were co-transfected into a 293FT donor cell line by using Lipofectamine 2000 (Invitrogen) to produce a replication-incompetent lentivirus. Lentivirus containing pre-miRNA cassettes or control (scramble miRNA sequence) were used to transduce the human breast cancer cell lines T47D and MCF7 to produce three *plakoglobin-*knockdown and control cell lines.

### Real-time PCR

Total RNA was extracted with Trizol (Invitrogen), before reverse transcription by using superscript III (Invitrogen); the resulting cDNA was used as a template for real-time quantitative PCR. Cells from three separate *in vitro *experiments or five tumors per *in vivo *group were analyzed separately. Relative mRNA expression of *plakoglobin *(Hs00984034_m1; Applied Biosystems), *e-Cadherin *(Hs1013953_m1), or *NM23-H1 *(Hs00264824_m1) were compared with the housekeeping gene *glyceraldehyde-3-phosphate dehydrogenase *(*GAPDH*; Hs99999905_m1) by using an ABI 7900 PCR system (Perkin-Elmer, Applied Biosystems) and Taqman universal master mix (Applied Biosystems). Relative mRNA was determined by using the formula 2^-ΔCT ^(CT; cycle threshold) where ΔCT = CT (target gene) - CT (*GAPDH*).

### Western blotting

Protein was extracted from pooled cells from three separate *in vitro *experiments and tumor samples from individual mice by using a Mammalian cell lysis kit (Sigma-Aldrich, Poole, UK). Ten micrograms of protein was run on a 10% polyacrylamide gel and transferred onto an imibilon-P nitrocellulose membrane (Millipore). Nonspecific binding was blocked with 1% casein (Vector Laboratories) before incubation with mouse monoclonal antibodies specific for gamma-catenin [15F11] (1:500), e-cadherin [HECD-1] (1:1,000), or GAPDH [6C5] (1:1,000) for 16 hours at 4°C (all primary antibodies were from AbCam, Cambridge, UK). Secondary antibody was sheep anti-mouse horseradish peroxidase (HRP; 1:15,000), and HRP was detected with the Supersignal chemiluminescence detection kit (Pierce). Band quantification was carried out by using Quantity One software (Bio-Rad, Hemel Hempstead, UK) and normalized to RNA polymerase.

### Immunohistochemistry

Immunohistochemistry was performed on 4% formalin-fixed cells grown on chamber slides. Primary antibodies rabbit anti-γ catenin (2309s, 1:500 dilution; New England Biolabs), rat anti-e-cadherin (DECMA-1, 1:50; AbCam), and rabbit anti- β-catenin (E247, 1:50; AbCam). Cells were subsequently incubated with biotin anti-rabbit (for γ-catenin and β-catenin at 1:100) and biotin anti-ray (for e-cadherin at 1:100). Visualization followed incubation with fluorescence avidin (Vector A-2001) at 1:250.

### nalysis of prometastatic parameters *in vitro *

All cell lines were maintained in RPMI-1640 supplemented with 10% FCS (Gibco Invitrogen, Paisley, UK) and 50 mg/ml blasticidin, 24 hours before experimental protocol cells were transferred to RMPI-1640 supplemented with 10% FCS in the absence of blasticidin. All experiments were carried out in triplicate and repeated 3 times. Cell proliferation was monitored every 24 hours for 96 hours by cell counting with a 1/400 mm^2 ^hemocytometer (Hawksley, Lancing, UK), and a sample of cells was taken at each time point for Western blotting and rt-PCR to check the stability of the siRNA knockdown.

Cell-cell adhesion was monitored by using two methods: (a) the ability of cells to form three-dimensional spheroids after seeding of 2,000 cells into a semisolid medium. Medium consisted of 1.5% agarose (Invitrogen) in RPMI media supplemented with 1% fungizone (Sigma-Aldrich) and 1% streptomycin (Sigma-Aldrich). The spheroid area was calculated 10 and 21 days after seeding by using ImageJ software (ImageJ v1.44; National Institutes of Health, Bethesda, MD, USA); and (b) the ability of cells to bind to a confluent monolayer of the corresponding cell type; cells were cultured to form a confluent monolayer, and 20,000 cells of the same type (*plakoglobin *knockdown or control) were labeled with 1 n*M *DiD (Invitrogen, Molecular Probes) for 30 minutes in serum-free media before being seeded onto the equivalent confluent cells. Two hours after co-culture, media containing floating cells were discarded, and adherent cells were trypsinized. The number of DiD-labeled cells that had adhered to nonlabeled cells of the same cell type was analyzed at 644 n*M *with flow cytometry.

Tumor cell invasion was assessed by using 6-mm Transwell plates with an 8.0-μm pore size (Costar; Corning Incorporated, Corning, NY, USA) either uncoated or coated with basement membrane matrix (Matrigel; Invitrogen). Cells were seeded into the inner chamber at a density of 2.5 × 10^5 ^and 5 × 10^5 ^per assay in RPMI without FCS, and RPMI supplemented with FCS was added to the outer chamber. Twenty-four hours after seeding, cells were removed from the top surface of the membrane, and cells that had invaded through the pores were stained with hematoxylin and eosin. Invasion was calculated as the percentage of cells that invaded through basement membrane compared with cells that had moved to the underside of uncoated plates. Numbers of cells were counted by using a DMRB microscope (Leitz, Germany) and OsteoMeasure XP v1.2.0.1 program (Osteometrics, Decatur, GA, USA).

Migration of tumor cells was investigated by analyzing wound closure: Cells were seeded onto 0.2% gelatin; once confluent, 10 µg/ml mitomycin C was added, and a 50-μm scratch made across the monolayer. The percentage of wound closure was measured at 24, 48, and 72 hours. To monitor migration of individual tumor cells, a 50-μm scratch was made across the monolayer of confluent cells. Images were taken every hour for 72 hours by time-lapse microscopy by using a CTR7000 inverted microscope; images were captured by using LAS-AF v2.1.1 software (Leica Applications Suite; Leica Microsystems, Wetzlar, Germany) and analyzed by ImageJ software (ImageJ v1.44; National Institutes of Health).

### Effects of altered reduced *plakoglobin *expression on tumor growth and metastases *in vivo *

#### Analysis of tumor growth *in vivo*

17β estradiol pellets (Innovative Research of America) were implanted subcutaneously into 50, 11-week-old female balb/c nu/nu mice (Harlan, Shardlow, UK). Seven days after the pellet had been implanted, 1 × 10^5 ^MCF7 3c-3 or control cells (n =15 per group) or 1 × 10^5 ^T47D 2A-4 or control cells (*n *= 10 per group) in 20 μl (33% Matrigel: 66% PBS) were inoculated into the fifth and 10^th ^mammary fat pads. Tumor volume was measured twice per week by using calipers. For differences in tumor growth, 10 mice per group were culled by cervical dislocation 32 or 34 days after tumor cell implantation. For analysis of circulating tumor cells, five mice per group bearing MCF7 3c-3 or control cells were culled once tumors were of equal size (1 cm^3 ^). Tumors were placed in cell-lysis buffer (MCL-1KT mammalian cell-lysis kit (Sigma, Poole, UK)), and protein was extracted according to the manufacturer's instructions. Experiments were carried out with UK Home Office approval under project license 40/2343.

#### Isolation of circulating tumor cells

Whole blood was analyzed from five mice per group bearing 1-cm^3 ^tumors from MCF7 3C-3 or control cells. Whole blood from three naïve mice and parental MCF7 cells were used for staining controls by using the protocol described in Ottewell *et al*. [20], with the exception that the primary antibody used was anti-human EpCAM antibody directly conjugated to phycoerythrin (PE) (Clone 1B7; 1:50; eBioscience, Hatfield, UK). PE-positive cells were plated directly into a 96-well tissue-culture plate (one well per group) (Nunc, Rochester, UK) on a MoFlow High performance cell sorter (Beckman Coulter, Cambridge, UK). PE fluorescence was detected with a 555LP dichroic long-pass and a 580/30-nm band-pass filter. Acquisition and analysis of cells was performed by using Summit 4.3 software. After sorting, cells were cultured in RPMI medium supplemented with 10% FCS.

### Microcomputed tomography imaging

Microcomputed tomography analysis of tibiae and femurs were carried out by using a Skyscan 1172 x-ray-computed microtomograph (Skyscan, Aartselaar, Belgium) equipped with an x-ray tube (voltage, 49 kV; current, 200 μA) and a 0.5-mm aluminum filter. Pixel size was set to 4.37 μm, and scanning was initiated from the top of the proximal tibia or the distal femur. For each sample, 275 cross-sectional images were reconstructed with NRecon software (version 1.4.3, Skyscan). After reconstruction, the volume of interest was defined on the two-dimensional acquisition images by using a hand-drawing tool. Trabecular bone volume fraction (BV/TV), the ratio of the volume of bone present (BV) to the volume of the cancellous space (TV), was calculated for 1 mm of the bone, starting 0.2 mm from the growth plate. 3D modeling and analysis were performed by using CTAn (version 1.5.0.2) and CTvol (version 1.9.4.1) software (Skyscan).

### Statistical analysis

Statistical analysis was carried out by using analysis of variance (ANOVA) followed by the Dunnett two-sided multiple comparison test. All *P *values are two-sided.

## Results

### *Plakoglobin *expression in parental cell lines

**Figure 1:**
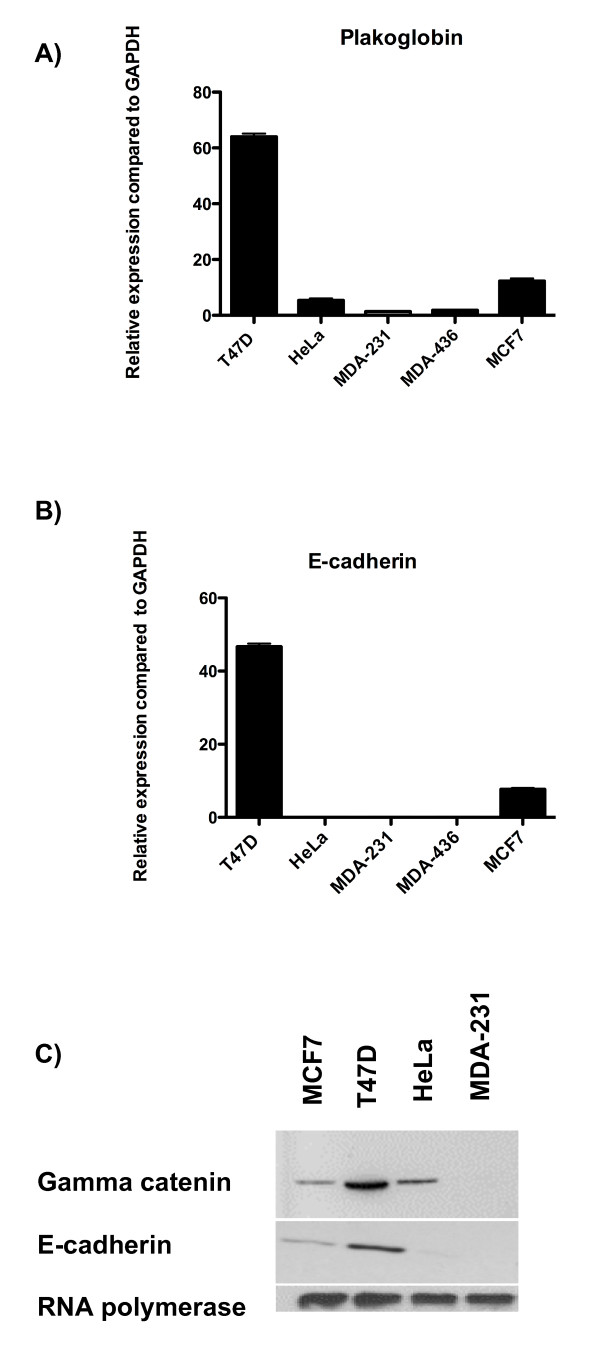
**Comparative expression of adhesion molecules**. Relative expression of **(A) ***plakoglobin *and **(B) ***e-cadherin *compared with *GAPDH *± SEM for T47D, HeLa, MDA-MB-231, MDA-MB-436, and MCF7 cells, as assessed with real-time PCR. **(C) **Western blots showing protein expression of γ-catenin, e-cadherin, and RNA polymerase from MCF7, T47D, HeLa, and MDA-MB-231 cells.

The main aim of this study was to investigate whether expression of the cell-adhesion molecule *plakoglobin *plays a role in the dissemination of breast cancer cells into the circulation. We first compared the expression of *plakoglobin *in weakly metastatic MCF7, nonmetastatic T47D, and highly metastatic HeLa, MDA-MB-436, and MDA-MB-231 cell lines [13-16,21-24]. Real-time PCR analysis showed significantly higher levels of *plakoglobin *in MCF7 and T47D breast cancer cells compared with HeLa, MDA-MB-231, or MDA-MB-426 (*P *< 0.0001 for T47D cells and *P *< 0.005 for MCF7 cells compared with HeLa, MDA-MB-231, or MDA-436) (Figure 1A). Furthermore, significantly higher levels of *plakoglobin *were detected in T47D cells compared with MCF7 (*P *< 0.005). In all cell lines tested, *plakoglobin *gene expression correlated with γ-catenin protein expression (Figure 1C).

The protein product of *plakoglobin*, γ-catenin, complexes with e-cadherin to form intercellular bonds; we therefore examined the gene and protein expression of e-cadherin in the highly metastatic versus weakly and nonmetastatic cell lines. Real-time PCR and Western blot analysis showed high levels of e-cadherin in T47D and MCF7 cells, whereas this gene was undetectable in HeLa, MDA-MB-231, and MDA-MB-436 cells (Figure 1B). Similar to *plakoglobin *expression, significantly less *e-cadherin *was detected in MCF7 cells compared with T47D (*P *< 0.005). As both *plakoglobin *and *e-cadherin *were detectable in MCF7 and T47D cells, these cell lines were selected as models for investigating the effects of reduced *plakoglobin *on parameters associated with metastases after targeted miRNA knockdown.

### Effects of miRNA transfection on *plakoglobin *expression

**Figure 2:**
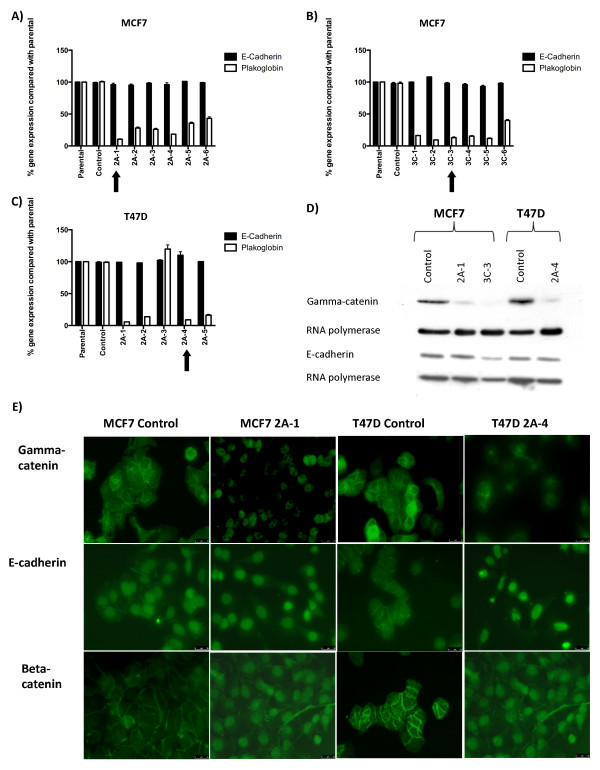
**Relative expression of *plakoglobin *and *e-cadherin *compared with *GAPDH *± SEM before and after siRNA knockdown**. **(A) **Scramble sequence or miRNA cassette 2 in MCF7 cells; **(B) **scramble sequence or miRNA cassette 3 in MCF7 cells; and **(C) **scramble sequence or miRNA cassette 2 in T47D cells. **(D) **Western blots showing γ-catenin and e-cadherin expression after transfection with scramble sequence or miRNA cassettes 2 and 3. **(E) **Immunohistochemical staining for γ-catenin, e-cadherin, and β-catenin (green). In the control cells, γ-catenin, e-cadherin, and β-catenin are expressed on the cell surface clearly demarcating the cell-cell junctions. In the knockdown lines, γ-catenin staining is reduced, and e-cadherin and β-catenin are detected in the nucleus and the cytoplasm.

To produce T47D and MCF7 cells with reduced levels of *plakoglobin*, pre-miRNA expression cassettes were designed to target the *plakoglobin *gene, and control cells were produced by using scramble sequence. Pre-miRNA cassettes were transfected into MCF7 and T47D cells, and six clones from each transfection were tested for *plakoglobin *expression. Transfection with scramble sequence had no effect on *plakoglobin *expression; however, transfection with miRNA 2 resulted in a 58% to 81% reduction in *plakoglobin *expression in MCF7 cells (Figure 2A) and a 0 to 90% reduction in expression in T47D cells (Figure 2C). Transfection miRNA cassette 3 resulted in a 55% to 83% reduction in *plakoglobin *expression in MCF7 cells (Figure 2B). MCF7 clone 2A-1 from cassette 2 and clone 3C-3 from cassette 3 showed a >80% reduction in *plakoglobin*, and T47D clone 2A-1 showed a 90% reduction in *plakoglobin *compared with scramble sequence; therefore, these clones were selected for use in the subsequent experiments. In all cases, the reduction in *plakoglobin *expression was translated into reduced γ-catenin levels (Figure 1D), with high levels of γ-catenin detected on the cell membrane of control MCF7 and T47D cells and reduced staining detected on the cell membranes of *plakoglobin-*knockdown cell lines (T47D cells shown in Figure 1E).

Real-time PCR analysis showed that knockdown of *plakoglobin *had no effect on total *e-cadherin *(Figure 2A, B, C). However, protein levels were decreased by 6% in MCF7 2A-1 and 6.5% in 3C-3 cells, whereas e-cadherin was increased by 13% in T47D 2A-4 cells, when normalized to RNA polymerase, compared with control (Figure 2D). Importantly, knockdown of *plakoglobin *resulted in the translocation of the cell-cell adhesion proteins e-cadherin and β-catenin from the cell membrane into the nucleus and cytoplasm (Figure 2E).

### Reduced expression of *plakoglobin *increases proliferation

**Figure 3:**
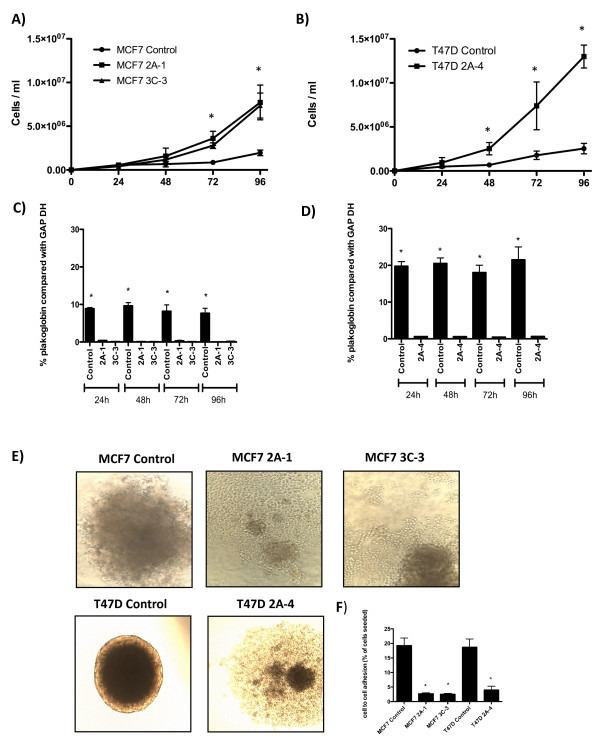
**Growth curves of control and knockdown cells**. Mean ± SEM for **(A) **MCF7 and **(B) **T47D control and *plakoglobin *knockdown cells. **(C, D) **Relative expression of *plakoglobin *over the 96-hour time course of the cell-proliferation experiment in control and knockdown MCF7 and T47D cells, respectively. **(E, F) **The effects of *plakoglobin *expression on tumor cell adhesion. (E) Spheroid formation and (F) the percentage of cells adhering to a confluent monolayer of cells of an identical cell type after 2 hours of co-culture. Data shown are expressed as mean ± SEM (*P < 0.005, by one-way ANOVA followed by the Dunnett two-sided multiple comparison test).

Reduced expression of *plakoglobin *resulted in a significant increase in proliferation of both MCF7 and T47D cells. In MCF7 cells a >80% reduction in *plakoglobin *led to a 2.5-fold increase in proliferation at 72 hours and a threefold increase at 96 hours compared with scramble sequence, with no significant difference between miRNA clones detected (*P *< 0.005 at 72 hours and *P *< 0.001 at 96 hours for 2A-1 and 3C-3) (Figure 3A). In T47D cells in which *plakoglobin *expression was reduced by 90%, proliferation was further increased with a twofold increase in numbers of cells at 48 hours (*P *< 0.005), a threefold increase at 72 hours (*P *< 0.005) and a fourfold increase at 96 hours (*P *< 0.0001) compared with control (Figure 3B). Furthermore, T47D 2A-4 cells showed significantly increased proliferation compared with control than did MCF7 2A-1 or 3C-3 clones (*P *< 0.005).

To ensure that miRNA knockdown was stable over time, the levels of *plakoglobin *in tumor cells were analyzed at 24, 48, 72, and 96 hours for each cell-proliferation study. Figure 1C and 1D show that *plakoglobin *is constitutively reduced in MCF7 and T47D miRNA knockdown cells compared with control, and these cells were used in the subsequent experiments.

### Reduced *plakoglobin *reduces cell-cell adhesion

A key early step in the metastatic process is the breaking away and dissemination of cells from the primary tumor mass. To investigate the effect of *plakoglobin *expression on cell-cell adhesion, we analyzed the ability of control and knockdown cell lines to form three-dimensional spheroid structures in agar. Figure 3E shows control MCF7 cells growing as spheroids on agar, demonstrating that these cells were able to form extensive bonds with surrounding cells. Control T47D, which expressed sixfold more *plakoglobin *than did control MCF7 cells (see Figure 1A), grew as a much tighter spheroid. Reducing *plakoglobin *levels resulted in a complete loss of spheroid formation in MCF7 cells; both 2A-1 and 3C-3 cells grew as monolayers across the surface of the agar, indicating that these cell lines no longer form strong intercellular bonds. Similarly, reducing *plakoglobin *in T47D cells resulted in decreased cell-cell adhesion, with 2A-4 cells growing in a less-compact spheroid formation than control cells and cells at the edge of the spheroid growing in a monolayer. The reduced cell-cell adhesion after loss of *plakoglobin *was confirmed by measuring the ability of DiD-labeled control and knockdown cells to adhere to a confluent cell monolayer of the corresponding cell type. Measurement of the percentage of cells binding to a monolayer after 2 hours (Figure 3F) showed that 19.23% ± 2.6% of DiD-labeled MCF7 control cells bound to unlabeled control cells, compared with 2.71% ± 0.30% MCF7 2A-1 cells binding to unlabeled 2A-1 cells and 2.54% ± 0.26% 3C-3 cells binding to unlabeled cells. In addition, T47D control cells were significantly more adhesive than T47D 2A-4 cells; 19.01% ± 2.79% of control cells adhered to a monolayer of the same cell type compared with 4.21% ± 1.06% of 2A-4 cells (*P *< 0.005), demonstrating that reduced expression of *plakoglobin *significantly reduces cell-cell adhesion.

### Effects of *plakoglobin *expression on tumor cell invasion and migration

**Figure 4:**
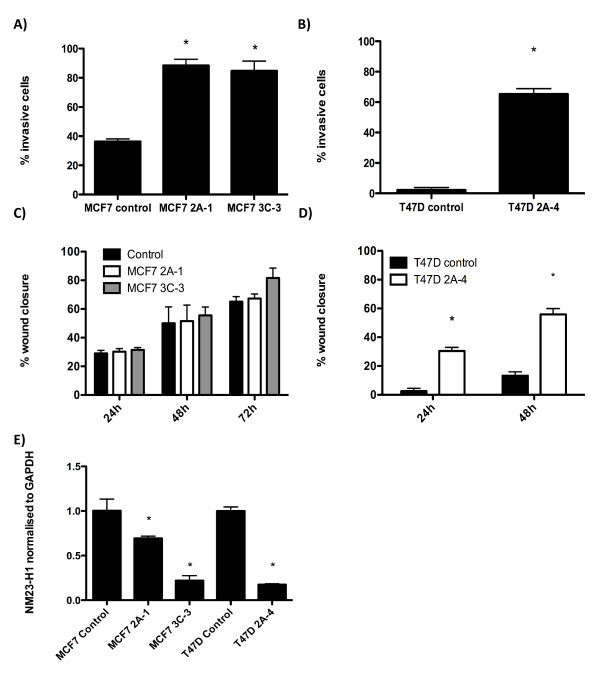
**Effects of *plakoglobin *expression on tumor cell invasion, migration, and expression of the tumor-suppressor gene *NM23-H1***. **(A, B) **Histograms of mean ± SEM percentage of MCF7 (A) and T47D (B) tumor cell invasion through a basement membrane matrix (Matrigel)-coated membrane compared with an uncoated membrane. Histograms showing mean ± SEM percentage of wound closure by MCF7 cells 24, 48, and 72 hours after scratch **(C)**, and T47D cells 24 and 48 hours after scratch **(D)**. **(E) **Expression of the tumor-suppressor gene, *NM23-H1*, in control and *plakoglobin-*knockdown MCF7 and T47D cells. (*P < 0.005, by one-way ANOVA followed by the Dunnett two-sided multiple-comparison test).

As invasion of cancer cells into the surrounding tissue and vasculature is an essential step for initiation of metastasis, we next investigated whether altering *plakoglobin *levels affects invasion through a basement membrane. Reducing expression of *plakoglobin *significantly increased MCF7 and T47D tumor-cell invasion through 8-µm pores in a Matrigel-coated membrane at 24 hours: 89.8% of MCF7 2A-1 cells and 86.5% of MCF7 3C-3 cells that migrated trough an uncoated membrane also invaded through Matrigel compared with 36.6% of MCF7 control cells (*P *< 0.001 for both knockdown cell lines compared with control). Similar proinvasive effects were also observed in *plakoglobin-*knockdown T47D cells: 63.6% of T47D 2A-4 cells invaded through Matrigel compared with just 1.3% of T47D control cells (*P *< 0.001; 2A-4 versus control) (Figure 4B). In the absence of a basement membrane, *plakoglobin-*knockdown cells were significantly more migratory than were control cells (250 ± 50.4 MCF7 2A-1 cells and 248 ± 54.2 MCF7 3C-3 cells migrated through 8-µm pores compared with 1.3 ± 2.1 MCF7 control cells) over a 24-hour period. These data imply that reduced expression of *plakoglobin *promotes cell migration toward a chemotactic agent.

As knockdown cells had increased ability to invade through an artificial basement membrane compared with control, we used a wound-healing assay to investigate whether *plakoglobin *expression altered cell motility in the absence of a chemotactic agent. No significant differences in cell motility were observed between MCF7 control and *plakoglobin-*knockdown cells over a period of 72 hours (Figure 4D), with cell tracking showing individual cells moving at an average of (23 ± 9 μm/h) for control; (27 ± 8 μm/h) for 2A-1 and (29 ± 9 μm/h) for 3C-3. In contrast, significantly increased cellular movement was observed in T47D 2A-4 cells compared with control; after 24 hours, 29.6% ± 3.2% of the wound was closed in 2A-4 cells compared with 3.3% ± 2.4% for control (*P *< 0.0001), increasing to 57.8% ± 4.2% in 2A-4 and 16.7% ± 3.5% in control cells after 48 hours (*P *< 0.0001; Figure 4E). Average cell movement measured by individual cell tracking was (11.5 ± 4.8 μm/h) for control and (18.6 ± 8.4 μm/h) for 2A-4 cells, clearly demonstrating that loss of *plakoglobin *expression is associated with a significant increase in cell motility, potentially leading to increased tumor cell spread.

### Effects of *plakoglobin *on expression of the tumor-suppressor molecule *NM23-H1*

Reduced expression of *NM23-H1 *has been shown to disrupt cell-cell adhesion mediated by e-cadherin, resulting in β-catenin nuclear translocation [25]. Furthermore, silencing of this gene promotes cellular scattering, motility, and extracellular matrix invasion [26]. We therefore investigated the effects of *plakoglobin *knockdown on expression of *NM23-H1*. As shown in Figure 4E reducing expression of *plakoglobin *by >80% resulted in a significant decrease of *NM23-H1 *in MCF7 2A-1 cells (*P *< 0.005) and T47D 2A-4 (*P *< 0.005) cells. In contrast, expression of *NM23-H1 *was not significantly decreased in MCF7 3C-3 cells (*P *= 0.08 compared with control), implying that prometastatic effects seen after *plakoglobin *knockdown may not be due to reduced expression of *MN23-H1*.

### Effects of *plakoglobin *on tumor cell growth and dissemination *in vivo *

**Figure 5:**
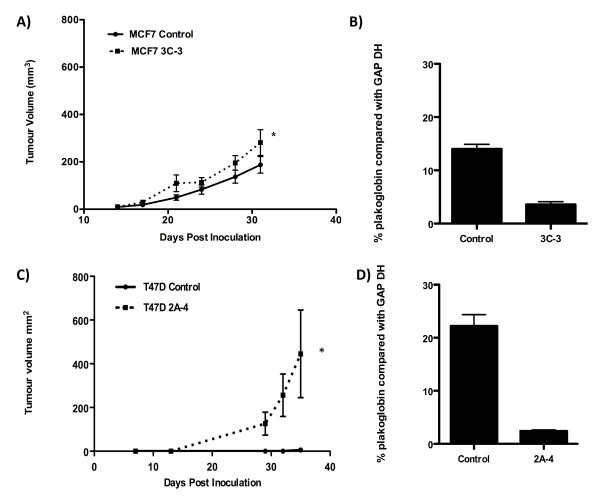
**Effects of *plakoglobin *expression on tumor growth *in vivo***. Ten Balb/c nude mice per group were inoculated with 1 × 10^5 ^MCF7 control or MCF7 3C-3 cells **(A) **or 1 × 10^5 ^T47D control or T47D 2A-4 **(B) **into the fifth and tenth mammary fat pads. Tumors were measured twice per week, and all animals were killed 32 (MCF7) or 34 (T47D) days after tumor implantation. Data show mean ± SEM tumor volume over time. **(C) ***Plakoglobin *expression in excised MCF7 control and 3C-3 tumors. **(D) ***Plakoglobin *expression in excised T47D control and 2A-4 tumors. PCR data are shown as a percentage of GAPDH. (*P < 0.05, by one-way ANOVA, followed by the Dunnett two-sided multiple comparison test).

As the *in vitro *data showed that reduced *plakoglobin *expression increased the prometastatic phenotype of MCF7 and T47D cells, we next investigated the effects of reduced *plakoglobin *on tumor cell growth and dissemination into the circulation *in vivo*. miRNA knockdown of *plakoglobin *resulted in a significant increase in MCF7 and T47D tumor growth in the mammary fat pads of nude mice (Figure 5A and 5C). At 32 days after inoculation of tumor cells, MCF7 3C-3 tumors measured 281.43 ± 54.31 mm^3^, and control tumors measured 184.21 ± 34.93 mm^3 ^(*P *< 0.001), with *plakoglobin *expression accounting for 30.7% of the total variance. Furthermore, mammary fat pad inoculation of T47D cells resulted in a 55-fold increase in growth of T47D 2A-4 cells compared with control cells (Figure 5C). Real-time PCR analysis of tumors after extraction confirmed that *plakoglobin *expression remained suppressed in knockdown cells *in vivo *(Figure 5B and 5C).

**Figure 6:**
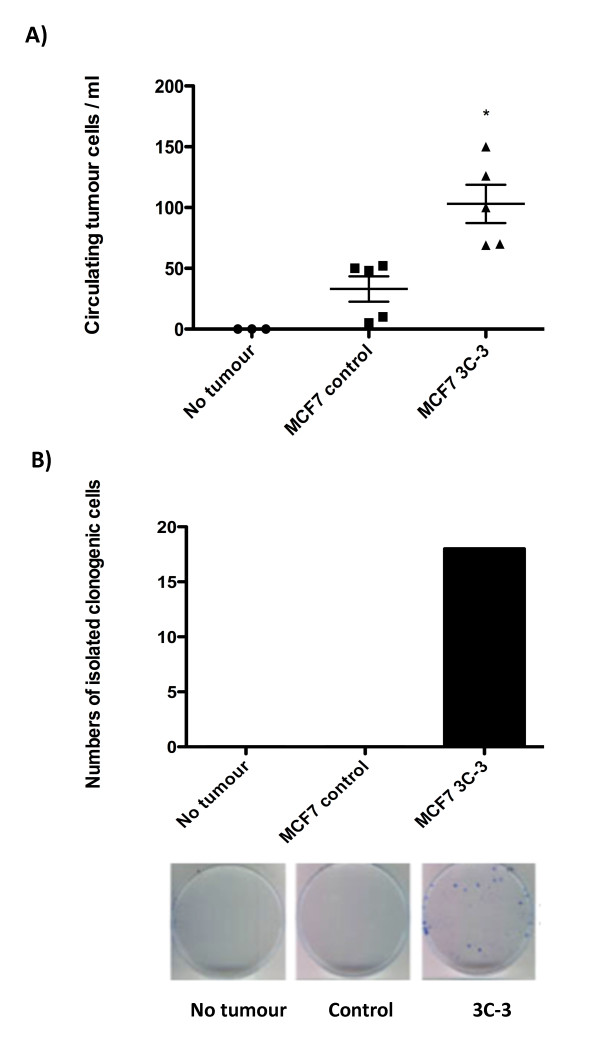
**Effects of *plakoglobin *expression on tumor cell shedding into the bloodstream**. **(A) **Numbers of circulating tumor cells per milliliter of blood isolated from five mice bearing control MCF7 cells and five mice with MCF7 3C3 tumors. Tumor cells were isolated from mice with equal tumor volume (1 cm^3 ^per mouse), and non-tumor-bearing mice were used as a control. Cells from each group were sorted into one well of a 96-well plate, and **(B) **the numbers of cells isolated from the circulation with clonogenic potential are shown. (*P < 0.005, compared with MCF7 control cells, by one-way ANOVA followed by the Dunnett two-sided multiple-comparison test).

MCF7 cells have previously been shown to home to and grow in mouse long bones when inoculated directly into the bloodstream [27]. We therefore analyzed the long bones of tumor-bearing mice for evidence of metastatic tumor growth. Microcomputed tomography analysis of tibiae and femurs from mice bearing MCF7 cells confirmed that no evidence existed of osteolytic lesions or tumor-induced bone loss in any mice bearing mammary fat pad tumors. In the absence of late-stage metastases (tumor in bone), we measured the effects of altered *plakoglobin *expression on an early metastatic event by comparing the number of tumor cells in the bloodstream in mice bearing MCF7 control tumors with those in mice bearing MCF7 3C-3 tumors of equal volume (Figure 6A). Blood from tumor-free animals was used as a negative control. Mice bearing 1 cc MCF7 3C-3 tumors had >2.5 times more tumor cells per millilitre of blood than did mice with 1-cc MCF7 control tumors, implying that reduced *plakoglobin *increases tumor cell shedding from the primary site into the circulation. No colonies formed by day 7 after plating of the 165 EpCAM-positive cells isolated from MCF7 control samples or tumor-free controls (Figure 6B). In contrast, 19 colonies grew from a total of 522 cells isolated from MCF7 3C-3 blood samples, showing that the EpCAM-positive population isolated from animals with *plakoglobin *knockdown tumors contains increased numbers of viable tumor cells.

## Discussion

In this study, we established that loss of *plakoglobin *leads to significant changes in breast cancer cell phenotypes, indicative of increased capacity for invasive growth. Accumulating evidence suggests that adhesive interactions are critical in the process of metastatic tumor dissemination and that adhesion molecules can act as both positive and negative modulators of the metastatic process [reviewed in [28]]. The γ-catenin-e-cadherin complex promotes homotypic tumor cell adhesion, maintaining intercellular contacts that confine cells to the primary tumor site. Many studies have shown a correlation between reduced e-cadherin expression and increased metastasis both in laboratory models and in clinical samples; however, a direct functional role of γ-catenin in this process has not been shown [29-33]. Evidence from clinical studies suggests that reduced expression of *plakoglobin *in human breast cancers is associated with increased tumor progression and adverse clinical outcome [34-36]. Additionally, translocation of γ-catenin from the membrane to the cytoplasm has been shown in breast cancers with a more-aggressive phenotype [37]. Fitting with our hypothesis that reduced *plakoglobin *expression increases the metastatic phenotype of cancer cells, we observed very low levels of *plakoglobin *in the highly metastatic cancer cell lines: MDA-MB-231, MDA-MB-436, and HeLa, higher levels of *plakoglobin *in weakly metastatic MCF7 cells, and very high levels in nonmetastatic T47D cells. In accordance with previously published studies of human breast cancer biopsies, we found that cells with low levels of *plakoglobin *expressed low levels of *e-cadherin*, and significantly more *e-cadherin *was detectable in nonmetastatic cell lines. Interestingly, levels of *plakoglobin *and *e-cadherin *not only correlated with each other, but also appeared to correlate with the metastatic potential of the cell lines tested, implying that loss of this catenin-cadherin complex is directly involved in the metastatic process.

In the current study, we investigated the specific effects of reduced *plakoglobin *expression on tumor cell metastasis. Breast cancer metastasis is initiated by the transition of epithelial cancer cells to a mesenchymal phenotype. We demonstrated that miRNA silencing of *plakoglobin *causes translocation of intercellular adhesion proteins e-cadherin and β-catenin from the cell membrane to the nucleus and the cytoplasm; these processes are strongly associated with epithelial-to-mesenchymal transition [26,38,39]. These prometastatic changes have been linked with downregulation of the tumor suppressor *NM23-H1 *[25]. In the current study, *NM23-H1 *expression was reduced in T47D 2A-3 and MCF7 2A-1 cells; however, *NM23-H1 *was unaltered in MCF7 3C-3 cells, which were significantly more migratory and invasive than MCF-7 2A-1 cells. Therefore, it appears that although altering intercellular adhesion molecules can downregulate *NM23-H1*, other interacting factors may be necessary for silencing of this gene. Key events in initiation of metastasis include tumor cell proliferation, detachment of cells from the primary tumor, migration toward the vasculature, and invasion into the blood and lymphatic systems. Knocking down *plakoglobin *expression substantially increased the prometastatic potential of both cell lines, increasing proliferation, reducing intercellular adhesion, and increasing invasion. Tumor cell migration was also increased in T47D knockdown cells compared with control. Interestingly, alterations in metastatic phenotype were more pronounced in T47D than in MCF7 cells. These observations may be due to the differences in *plakoglobin *expression between the two cell lines: T47D miRNA knockdown cells express between 19- and 21-fold lower *plakoglobin *levels compared with control, whereas *plakoglobin *expression in MCF7-knockdown cells is six- to eightfold lower compared with MCF7 control. MCF7 2A-1 cells that show a sixfold reduction in *plakoglobin *compared with control are less invasive and migratory than MCF7 3C-3 cells, whose *plakoglobin *expression is reduced eightfold compared with control. These data strongly indicate that the level of *plakoglobin *expression within a breast cancer cell specifically correlates with its ability to undergo events involved in escape of tumor cells from the primary site.

For a metastatic tumor to grow at a distal site, tumor cells must be able to invade the circulatory system, survive in the bloodstream, and home to and colonize a specific metastatic site [23,28,40]. In accordance with our *in vitro *findings, *plakoglobin-*knockdown cells grew significantly faster than control cells when inoculated into the mammary fat pads of balb/c nude mice. In addition, increased numbers of EpCAM-positive tumor cells were isolated from whole blood in mice with MCF7 3C-3 tumors compared with controls, and the cells were viable and able to form colonies when grown in culture. These data indicate a higher degree of tumor cell dissemination into the circulation from mammary tumors with low levels of *plakoglobin *than from tumors expressing high levels of this gene. Despite high levels of circulating EpCAM-positive cells in mice with MCF7 3C-3 tumors, metastatic tumor growth was not detected in the bones of any animals. This finding may be due to MCF7 cells being unable to undergo the late stages of metastases (bone homing and colonization), although MCF7 cells have previously been used to induce breast cancer growth in bone after intracardiac inoculation [41]. The most likely explanation for this finding is that mice used in the current study were 10 to 12 weeks old when tumors were inoculated, and we and others have shown tumors do not take if animals that are older than 8 weeks. It is hypothesized that high bone turnover, as seen in young animals, is critical for human tumor metastasis to mouse bone [41,42]. We were unable to test this hypothesis in the mammary fat pads of 6-week-old animals, as the fat pads were too small to inoculate. It is also possible that any tumor metastases present in bone were undetectable because of the short period of these experiments (~5 weeks compared with 12 weeks required for metastatic lesions to develop in mouse bones after intracardiac inoculation of MCF7 cells) [42]. The effects of *plakoglobin *expression on tumor cell homing and colonization of distal organs clearly warrant further investigation and will form part of our future research strategy.

## Conclusion

In conclusion, this study is the first to show that reduced *plakoglobin *expression results in translocation of intercellular adhesion proteins e-cadherin and β-catenin from the cell membrane to the nucleus and cytoplasm, as well as increasing cell-cell detachment, invasion, and intravascular dissemination of breast cancer cells. Taken together, our results show that *plakoglobin *has a functional role in the metastatic process; it may therefore be a suitable prognostic marker for breast cancer metastasis and be considered a potential therapeutic target.

## Abbreviations

*E. coli: Escherichia coli*; FCS: fetal calf serum; GAPDH: glyceraldehyde 3-phosphate dehydrogenase; HRP: horseradish peroxidase; miRNA: microribonucleic acid; μCT: microcomputed tomography; PE: phycoerythrin; RNAi: interfering ribonucleic acid; ssRNA: single-stranded ribonucleic acid.

## Competing interests

The authors declare that they have no competing interests.

## Authors' contributions

IH was co-applicant on the grant providing the funds for this research, helped prepare the manuscript, and had significant academic input into this project. JW carried out the *in vitro *experiments and contributed to the data analysis, under the supervision of IH and PDO. FN carried out the first set of *in vivo *studies. AE designed the immunohistochemical protocols and performed the staining. HKB and DL carried out the third and fourth sets of *in vivo *studies and performed statistical analysis. IB developed the method of cell tracking used to analyze the movement of cells over time in this study. MJ was responsible for isolating circulating tumor cells from mouse whole blood. PDO was principal investigator on this study and was responsible for the original hypothesis, study design, final data analysis, and write-up. All authors read and approved the final manuscript.
