# Erratum to: Loss of *plakoglobin* promotes cell-cell contact, increased invasion and breast cancer cell dissemination in vivo

**DOI:** 10.1186/s13058-017-0835-4

**Published:** 2017-03-28

**Authors:** Ingunn Holen, Jacob Whitworth, Faith Nutter, Alyson Evans, Hannah K. Brown, Diane V. Lefley, Ivana Barbaric, Mark Jones, Penelope D. Ottewell

**Affiliations:** 1 Academic Unit of Clinical Oncology, Beech Hill Road, Sheffield, UK; 2 Centre for Stem Cell Research, Biomedical Sciences, Western Bank, University of Sheffield, Sheffield, UK; 3 Academic Unit of Clinical Oncology, CR-UK/YCR Sheffield Cancer Research Centre, University of Sheffield, Sheffield, S10 2RX UK

## Erratum

After the publication of this study [1] an error was detected in Fig. 2e. The same image was accidently used for beta-catenin staining of MCF7 2A-1 and T47D 2A-4. This error does not affect the findings or conclusions of the article. The corrected figure is shown below and we apologise for this mistake.

**Fig. 2:**
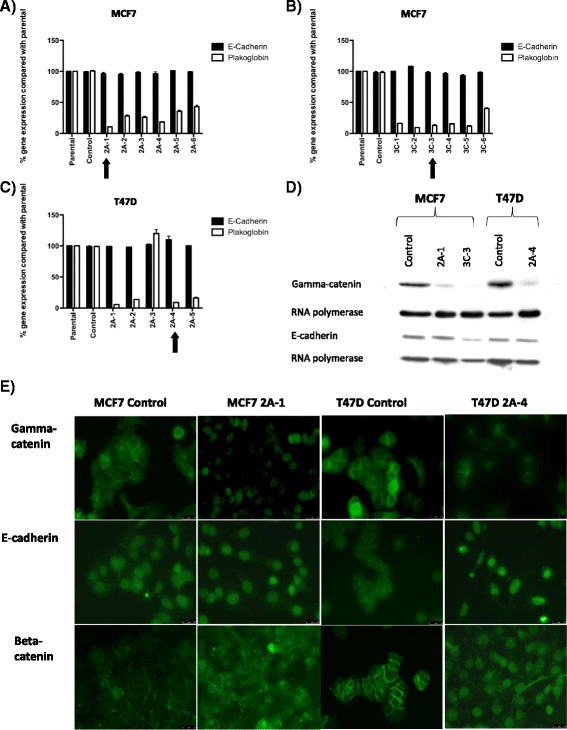
Shows relative expression of *plakoglobin* and *e-cadherin* compared with GAPDH ± SEM before and after siRNA knockdown with (**a**) scramble sequence or miRNA cassette 2 in MCF7 cells, (**b**) scramble sequence or miRNA cassette 3 in MCF7 cells, (**c**) scramble sequence or miRNA cassette 2 in T47D cells. **d** Are Western blots showing gamma catenin and E-cadherin expression following transfection with scramble sequence or miRNA cassettes 2 and 3. **e** Shows immunohistochemical staining for γ-catenin, e-cadherin and beta-catenin (*green*). In the control cells γ-catenin, e-cadherin and β-catenin are expressed on the cell surface clearly demarcating the cell-cell junctions. In the knock down lines, γ-catenin staining is reduced and e-cadherin and β-catenin is detected in the nucleus and the cytoplasm and β-catenin
